# Glycinergic transmission: glycine transporter GlyT2 in neuronal pathologies

**DOI:** 10.1042/NS20160009

**Published:** 2016-12-22

**Authors:** Francisco Zafra, Ignacio Ibáñez, Cecilio Giménez

**Affiliations:** Centro de Biología Molecular Severo Ochoa, Facultad de Ciencias, Consejo Superior de Investigaciones Científicas, Universidad Autónoma de Madrid, Madrid, Spain

**Keywords:** glycine, hyperekplexia, myoclonus, neuropathic pain, synaptic transmission, transporters

## Abstract

Glycinergic neurons are major contributors to the regulation of neuronal excitability, mainly in caudal areas of the nervous system. These neurons control fluxes of sensory information between the periphery and the CNS and diverse motor activities like locomotion, respiration or vocalization. The phenotype of a glycinergic neuron is determined by the expression of at least two proteins: GlyT2, a plasma membrane transporter of glycine, and VIAAT, a vesicular transporter shared by glycine and GABA. In this article, we review recent advances in understanding the role of GlyT2 in the pathophysiology of inhibitory glycinergic neurotransmission. GlyT2 mutations are associated to decreased glycinergic function that results in a rare movement disease termed hyperekplexia (HPX) or startle disease. In addition, glycinergic neurons control pain transmission in the dorsal spinal cord and their function is reduced in chronic pain states. A moderate inhibition of GlyT2 may potentiate glycinergic inhibition and constitutes an attractive target for pharmacological intervention against these devastating conditions.

## Introduction: the building of a glycinergic neuron

Glycine is present at rather low concentrations in all cells of an organism since it is one of the proteinogenic amino acids. However, relatively small subpopulations of neurons display a much higher cytoplasmic concentration of glycine and use it as a neurotransmitter. These are known as glycinergic neurons, a heterogeneous group of interneurons with variable morphological, electrical and neurochemical properties that, together with GABAergic neurons, balance the excitatory activity of glutamatergic pathways in the nervous system, thereby controlling fluxes of sensory and motor information. Glycinergic neurons were initially identified by immunohistochemistry with anti-glycine antibodies in glutaraldehyde fixed tissue, and were found scattered along diverse nuclei of the brainstem like the pontine, the reticular or the auditory nuclei, as well the cerebellum and the different layers of the spinal cord [1,2]. Despite this morphological and neurochemical heterogeneity, most glycinergic neurons share a common property: the presence in the cell surface of GlyT2, a reliable marker of these neurons [3]. A major reason for the limited understanding of the contribution of glycinergic neurons to neuronal circuits is probably the difficulty in identifying these neurons in living brain slices in order to perform electrophysiological recordings on them. To circumvent these difficulties and taking into account the clear association of GlyT2 to glycinergic neurons, Zeilhofer et al. [4] generated a transgenic mouse expressing the fluorescent protein EGFP under the control of a fragment of the GlyT2 promoter. In these mice, EGFP (enhanced green fluorescent protein) expression largely overlaps with GlyT2 immunoreactivity in axon terminals, and with glycine immunoreactivity in neuronal somata. EGFP is sufficiently bright to identify glycinergic neurons in fixed tissue and in living slices and, therefore, constitutes an excellent tool for investigating the physiological contribution of glycinergic neurons to central nervous system physiology (for instance, see [5–7]). Independent of the detailed anatomical distribution and the exquisite morphological details of glycinergic neurons revealed by EGFP, and even though some minor ectopic expression of the transgene was observed, it seemed clear that the segment of the promoter used in these mice contained the major regulatory elements for correct expression of the transgene and, consequently, this segment could be used to design novel mice expressing the recombinase Cre specifically in glycinergic neurons. Indeed, these mice have been generated, and the GlyT2-Cre mice are suitable for the selective targeting of floxed genes and transgenes in glycinergic interneurons of brainstem and spinal cord [6,8]. Data recently obtained in these mice are producing relevant information about the role of subpopulations of glycinergic neurons in processing motor and sensory information [6,9–12].

GlyT2 is a transporter protein of the SLC6 family of sodium- and chloride-dependent neurotransmitter transporters. This family includes an additional glycine transporter, GlyT1, as well as the transporters for GABA, dopamine, norepinephrine, serotonin, proline, taurine, creatine, betaine and other orphan transporters [13]. All of them are membrane glycoproteins with a structural arrangement of 12 transmembrane segments and work as secondary active transporters coupled to the Na^+^ gradient maintained by the Na^+^/K^+^ ATPase, a protein that is tightly associated with GlyT2 [14]. Cl^−^ is also required for the transport activity of GlyT2 and other SLC6 transporters. GlyT2, like other members of the family, traffics to the cell surface as homo-oligomers from the endoplasmic reticulum (ER). There, the folding process is assisted by chaperones like calnexin, and the correctly folded and oligomerized transporter is packed into COPII vesicles and transferred to the Golgi complex, where it is heavily glycosylated [15–17]. Then, GlyT2 is sorted to the plasma membrane in lipid rafts, microdomains where it interacts with proteins like neuronal plasma membrane Ca^2+^-ATPase (PMCA), and Na^+^/Ca^2+^-exchanger 1 (NCX1). Endogenous PMCA and NCX activities are necessary for GlyT2 activity and this modulation depends on lipid raft integrity [18]. In neurons, GlyT2 is concentrated at presynaptic elements in a process dependent on the PDZ-interacting motif located in its C-terminus [19]. The trafficking of the transporter continues with its endocytosis either for lysosomal degradation of for recycling in a process dependent on GlyT2 ubiquitination [20,21].

In general, it is thought that the main role of neurotransmitter transporters is the termination of neurotransmission by removing the neuroactive substrate from the synaptic cleft to presynaptic neurons or to adjacent glial cells. Initially, the localization of GlyT2 seemed to fit into this scheme since immunohistochemical studies revealed that GlyT2 is abundantly expressed in glycinergic terminals along the brain stem, the cerebellum or the spinal cord, that is, in areas where glycine is released from glycinergic neurons [22]. However, this role was questioned by observations performed in GlyT2 knockout mice that showed increased instead of decreased synaptic levels of glycine, and this was attributed to a lower release of glycine from presynaptic terminals [23]. Thus, the main function of GlyT2 seems to be the building of a stepped gradient of glycine through the presynaptic plasma membrane to supply enough glycine for presynaptic vesicle refilling, a process necessary to preserve quantal glycine content in synaptic vesicles [24]. By contrast, the termination of the glycinergic signal by neurotransmitter reuptake seems to be reserved primarily to GlyT1, which in the spinal cord and brainstem is expressed mainly in glial cells surrounding glycinergic synapses [22,25]. However, some evidence indicates that GlyT2 may also contribute to this reuptake function. For instance, the pharmacological blockade of GlyT2 in lamina X neurons of rat spinal cord slices increases glycinergic neurotransmission in the spinal cord [26]. Similarly, microdialysis perfusion of the lumbar dorsal spinal cord of rats detected an increase of the extracellular glycine concentration in the presence of a GlyT2 inhibitor [27], suggesting that in caudal regions of the CNS, glial GlyT1 and neuronal GlyT2 closely cooperate in the reuptake of extracellular glycine at inhibitory synapses.

Consistent with the idea of GlyT2 being a primary accumulator of glycine into presynaptic terminals, electrophysiological measures revealed that this transporter has a unique coupling to the driving force provided by the Na^+^ gradient. Thus, while most of the transporters of this family present a cation coupling stoichiometry of two Na^+^ transported with every neurotransmitter molecule (including GlyT1), GlyT2 is coupled to the electrochemical movement of three Na^+^, favoring the maintenance of a high glycine concentration gradient along the presynaptic membrane [28]. Recent structure–function studies have enhanced our current understanding of mechanisms of electrochemical coupling, studies that have been impelled by the determination of the crystal structures of two SLC6 transporters, the sodium-dependent bacterial leucine transporter, LeuT [29], and the *Drosophila* dopamine transporter, dDAT [30]. These transporters arrange the 12 transmembrane (TM) α-helices in a “shallow shot glass”-like structure with substrate- and ion-binding sites being located halfway across the membrane at the bottom of an extracellular-facing cavity. After binding, the transporter oscillates to an inward orientation releasing substrates and ions into the intracellular face of the membrane. Two Na^+^-binding sites have been identified in LeuT and dDAT that are conserved in GlyT2 and other SLC6 transporters [29–32]. Another potential binding site for Na^+^ was identified in a cavity of GlyT2 formed by Trp^263^ and Met^276^ in the third transmembrane domanin (TM3), Ala^481^ in TM6 and Glu^648^ in TM10 [33]. However, it is unclear if this represents the third Na^+^-binding site that energizes the transport of glycine.

In order to become glycinergic, a neuron not only requires GlyT2 on its surface but also a vesicular glycine transporter in the synaptic boutons [34]. This transporter, termed VIAAT or VGAT, belongs to the SLC32 family and uses the electrochemical proton gradient generated in synaptic vesicles by the H^+^-ATPase to accumulate glycine into the vesicular lumen [35]. However, VIAAT is not specific for glycine and also catalyzes the transport of GABA into synaptic vesicles. Indeed, the affinity for GABA is higher than that of glycine (IC_50_ ≈ 5 mM vs 25 mM) [36] and, as a consequence, uptake of glycine by VIAAT is rather inefficient unless glycine is enriched intracellularly through the aforementioned activity of GlyT2. Because most of the GlyT2 containing neurons in the spinal cord also express the GABA synthetizing enzymes GAD65 and/or GAD67, GABA and glycine accumulate and are co-released from the same presynaptic vesicles [37–39]. Nevertheless, while the cytoplasmic glycine:GABA ratio might be essential in determining the content of the vesicles, additional regulatory mechanisms seem to operate within the terminal to displace the competition in favor of one or the other neurotransmitter (experimental evidence reviewed in [40]). Furthermore, although GABA and glycine are simultaneous and continuously released from terminals throughout development, postsynaptic responses evolve with the developmental stage of the synapse. While in slices derived from young animals co-released neurotransmitters activate both postsynaptic GABA and glycine receptors (GABARs and GlyRs, respectively), in adult cells the quantal postsynaptic responses are either GlyR- or GABAR-mediated [41] suggesting a remodeling of postsynaptic receptors. Nevertheless, it is still unclear whether co-release is maintained after the postnatal shift towards pure GABA or pure glycinergic transmission. Further refinement of the crosstalk between the co-released transmitters may involve other mechanisms like presynaptic GABA_B_ receptors [42].

Global genetic ablation of VIAAT results in perinatal lethality due elimination of GABAergic and glycinergic inhibitory neurotransmission and, consequently, the defective functioning of motoneurons responsible of the cardio-respiratory functions [43]. Similarly, conditional deletion of VIAAT restricted to glycinergic neurons using the above mentioned GlyT2-Cre mice resulted in pups that were not viable at birth due to respiratory failure [11].

## GlyT2 in neuronal pathologies

### Movement diseases: hyperekplexia

Glycinergic interneurons in the brainstem and spinal cord control diverse motor functions like locomotion or respiration. They provide synaptic input to motoneurons of the ventral spinal cord or the respiratory-related motor nuclei, thereby shaping the signaling to motoneurons [44,45]). Central glycinergic synaptic activity also plays a vital role in regulating motoneuron morphology and glutamatergic central synaptic inputs to these neurons during late embryonic development [46]. The activity of GlyT2 is essential for maintaining diverse aspects of the motor function. For instance, the GlyT2 inhibitor ALX-1393 inhibits the spontaneous activity of motoric spinal networks by inducing glycinergic tonic currents in the spinal ventral horn [47]. Glycinergic neurons also control a brainstem reflex, termed the startle reflex, which places the body in defensive posture following unexpected stimuli such as a sudden noise, touch or visual input. The startle response involves proximal and distal muscles and produces brief, shock-like movement comprising grimacing, arm abduction and flexion of the neck, trunk, elbows, hips and knees. Dysfunction of inhibitory glycinergic neurotransmission disrupts the startle reflex causing a rare neurological disorder called hyperekplexia (HPX, OMIM 149400), characterized by neonatal hypertonia and exaggerated startle responses, followed by muscle stiffness [48]. The disease can result in sudden death due to infantile episodes of apnea. Nevertheless, the majority of patients survive with treatment with benzodiazepines, although they may suffer accidental falls, that could result in serious injuries, throughout their lives [48]. The first identified and most frequent causes of HPX are several missense and nonsense mutations in the GlyR α1 subunit gene (GLRA1) [49]. Although these mutations have been traditionally considered as an example of postsynaptic disease, recent evidence also supports a role of presynaptic homomeric GlyRs in the pathogenesis of some GLRA1 mutations [50]. Rare cases of HPX are also associated with mutations in other components of the glycinergic synapse, i.e. GlyR β subunit (GLRB), collybistin (ARHGEF9) and gephyrin (GPHN), three proteins involved in the postsynaptic arrangement of GlyRs [49]. The second most frequent cause of HPX are mutations in SLC6A5 gene, which encodes GlyT2. The first evidence for this association was obtained after inactivation of the mouse gene, which generated a complex postnatal neuromotor phenotype that also showed some clinical signs of HPX [23]. Similarly, a spontaneous mutation in mouse Slc6a5, caused by a MusD retrotransposon insertion, also results in handling-induced spasms at 5 days of age, and mice that only survive for 2 weeks, presenting alterations in neuromuscular junction maturation [51]. Several missense, nonsense and frameshift mutations in the SLC6A5 gene cause HPX in humans [52–54], as well as related congenital neurological disorders in dogs [55], and cattle [56]. Nonsense and frameshift mutations produce inactive truncated transporters that are retained in the endoplasmic reticulum (W151X, R191X, Y297X, Y377X, R439X, V432F+fs97, Q630X, P108L+fs25, L198R+fs123, S489F+fs39, I665K+fs1). However, missense mutations generate proteins that in general reach the plasma membrane but contain modifications in residues important for the catalytic cycle, like the binding sites for Na^+^ (N509S, A275T), Cl^−^ (S513I), or glycine (W482R affects directly while A275T and E248K affect indirectly), and others probably interfere with the folding or conformational changes that occur during the translocation cycle (L237P, L243T, T425M, Y491C, F547S, Y656H, G657A) [52–54]. Most of the described SLC5A5 mutations have an autosomal recessive inheritance and the parental carriers are typically asymptomatic indicating that dominant negative effects are not a common mutational mechanism [52]. Nevertheless, two dominant mutations have been described. One of them, S510R, affects GlyT2 intracellular trafficking and blocks the arrival of the transporter to the plasma membrane [54]. A detailed study of the pathogenic mechanisms in mutant S512R of the rat sequence (equivalent to the human mutation S510R) indicates that the presence of an arginine residue rather than serine provokes GlyT2 misfolding, enhances its association to the ER-chaperone calnexin, alters the association with the COPII component Sec 24D and, as a consequence, impedes the exit of the transporter from the ER [57]. The S512R mutant forms oligomers with wild-type GlyT2 causing its retention in the ER and therefore the dominant negative effect. Similarly, the mutation Y705C, found in eight individuals from Spain and the UK, has a dominant inheritance [58]. The introduced cysteine residue aberrantly interacts with the cysteine pair Cys^311^/Cys^320^ in the second external loop of GlyT2. This interaction impairs transporter maturation through the secretory pathway, reduces surface expression, and inhibits transport function. Although this effect seems to be not dominant on the trafficking of the wildtype GlyT2, the mutant protein alters the H^+^ and Zn^2+^ sensitivity of wildtype GlyT2 in a dominant negative manner, thereby affecting the neurochemical properties of the GlyT2 hetero-oligomers carrying wild type and mutant subunits [58].

Although severely decreased, glycinergic transmission is not completely absent in GlyT2-KO mice, suggesting that other routes of glycine uptake or *de novo* synthesis of glycine exist in presynaptic terminals. Indeed, recent studies indicate that mutations in the neutral amino acid transporter Asc-1 cause HPX-like dysfunctions in mice, and the corresponding gene (SLC7A10) is considered as a candidate gene for human HPX [59]. This transporter has been localized by immunohistochemistry in nerve terminals, although a detailed study about the nature of these terminals is still missing. It is also unclear how Asc-1 collaborates with GlyT2 and perhaps GlyT1 in controlling glycine fluxes in synapses.

### Sensory dysfunctions: neurogenic and inflammatory pain

While motor functions are rather concentrated in the ventral horn of the spinal cord, the dorsal horn represents a conveyor of sensory information and its circuitry is extensively remodeled in neuropathic pain states. Considerable evidence unequivocally supports the pivotal role of glycinergic neurons in these plastic changes. Moreover, since GlyT2 is a key component of the glycine fluxes, this transporter and GlyT1, have become candidate targets for pharmacological intervention to alleviate neuropathic and inflammatory pain.

**Figure 1:**
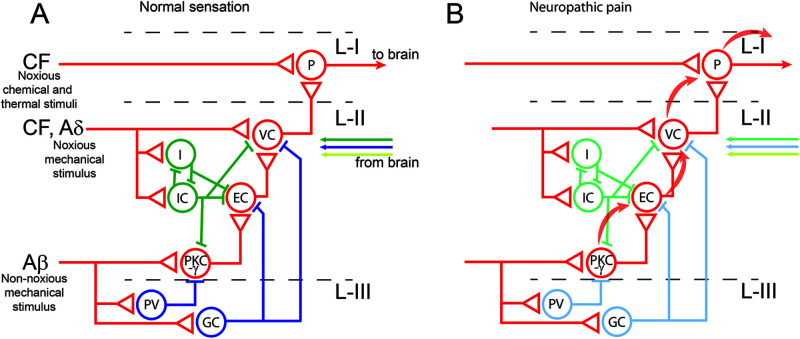
Circuits controlling pain transmission in the dorsal horn of the spinal cord. (**A**) Under normal physiological conditions, noxious and non-noxious sensory information reaches the dorsal spinal cord through different types of afferent fibres and is transmitted to supraspinal areas though projection neurons (P) of the dorsal horn lamina I (L-I). Non-noxious mechanical and thermal information cannot reach these neurons since it is filtered by several subpopulations of GABAergic (green circles; I, islet cells; IC*,* inhibitory central cells) and glycinergic neurons (blue circles), most of which are also GABAergic, residing in laminae II (L-II) and III (L-III) of the dorsal horn, as well as by inhibitory pathways descending from the brain. Several subpopulations of glycinergic neurons (GC) exist. One of them contains the marker parvalbumin (PV) and inhibits a subpopulation of excitatory interneurons involved in the transmission of non-noxious touch sensory information and that are characterized by the expression of PKCγ. (**B**) Lesions that induce neuropathic or inflammatory pain reduce the inhibitory tone (pale green and blue circles) and, then, innocuous signals can reach the projection neurons (pale red curved arrows) through a network of excitatory interneurons (red circles; EC, excitatory central cells; VC, vertical cells). Moderate inhibition of GlyT2 seems to restore glycinergic inhibition by increasing glycine levels in the neighborhood of these local excitatory circuits. The scheme is based on [61,62,67,71]

Detailed articles on the organization of this area of the nervous system and its remodeling in neuropathic pain conditions have been published recently [60–62]. In brief, four neuronal components contribute to these circuits (Figure 1A):

1. Primary afferent axons that originate from neurons located in the dorsal root ganglia and are classified according to their structure and type of information transported. The larger myelinated (Aβ) afferents transmit innocuous mechanical information, while most finely myelinated (Aδ) and unmyelinated (C) fibres are activated by noxious thermal or mechanical stimuli. Their central projections are organized in a very specific manner, with Aδ and C fibres branching mainly in the superficial layers I and II of the spinal dorsal horn while Aβ tactile afferents terminate in the deeper layers (III–V).
2. Projection neurons (cells with axons that travel directly to supraspinal nuclei), concentrated in lamina I and scattered through laminae III–VI. Their ascending axons form the anterolateral tract that targets the thalamus, periaqueductal grey matter, lateral parabrachial area and several nuclei in the medulla.
3. Interneurons (neurons with axons that remain within the spinal cord) represent more than 90% of the neurons in laminae I–III. About one-third of them are inhibitory (GABAergic, glycinergic or mixed) and the rest are excitatory (glutamatergic). In the superficial dorsal horn, i.e. in the termination area of nociceptors, purely GABAergic neurons dominate, whereas in deeper layers, where myelinated non-nociceptive neurons terminate, there is an enrichment in mixed GABA/glycinergic neurons.
4. Axons that descend from supraspinal areas are mainly monoaminergic and emanate from neurons in the raphe nuclei, the locus coeruleus and the pons. However, there is also an inhibitory projection from the ventromedial medulla that branches throughout the dorsal horn.

Regarding the inhibitory mechanisms that regulate nociception and chronic pain states, the initial gate control theory of pain proposed that the activity of these interneurons is controlled by electrical input from low-threshold mechanosensitive fibers to increase inhibition, and from high-threshold nociceptors to reduce inhibition [63]. Peripheral inflammation or nerve injuries initiate a functional reorganization of the dorsal horn circuitry, which leads to a net reduction in inhibitory tone and increased excitability in ascending pathways [61,64]. Although the molecular and cellular mechanisms underlying this process are unclear, disinhibition of glutamatergic neurons opens the gate to pathological pain signaling in superficial projection neurons of the spinal cord (Figure 1B). Glycinergic interneurons are key players in this process. For instance, a specific glycine receptor subtype (GlyR α3), which is enriched in lamina II of the dorsal horn, is inhibited by prostaglandin E2 released in inflammatory processes. The subsequent decrease in glycinergic activity apparently underlies central thermal and mechanical hypersensitivity, which develops within hours after induction of peripheral inflammation [65].

Similarly, removal of glycinergic inhibition with strychnine promotes the development of tactile allodynia, converting normally innocuous mechanical stimuli into painful sensation. In this condition, innocuous mechanical stimuli can activate superficial dorsal horn nociceptive specific neurons, neurons that do not normally respond to touch. This activation is mediated through a local circuit involving neurons expressing the gamma isoform of protein kinase C γ (PKCγ) [66]. PKCγ neurons, enriched in the inner part of layer II, are indeed controlled by a subset of mixed glycine/GABA interneurons containing parvalbumin (PV). Ablation of these neurons produced neuropathic pain-like mechanical allodynia, while activating PV neurons in nerve-injured mice alleviates mechanical hypersensitivity [67]. Consistently, another study performed in Cre-GlyT2 mice, where glycinergic neurons were ablated with exquisite regional precision in the deep dorsal horn layers by using spinal injections of viral particles driving the Cre-dependent expression of a neurotoxin, revealed the critical role of these neurons in processing noxious thermal and mechanical signals [9]. In this experimental system, silencing of glycinergic neurons induces thermal and mechanical sensitization and spontaneous pain. Conversely, pharmacogenetic stimulation of these neurons alleviated neuropathic hyperalgesia [9]. Also, inhibitory descending pathways from rostroventral medulla to lamina II inhibit mechanical nociceptive responses and these descending inhibitory mechanisms are suppressed in chronic pain states [68]. Altogether this evidence support the idea that pharmacological potentiation of glycine-mediated neurotransmission is an attractive possibility against neuropathic pain. Thus, the question is whether glycine transporter inhibitors increase or decrease the glycinergic tone in the spinal cord. Data indicate that inhibitors of both GlyT1 and GlyT2 reduce the abnormal pain sensations associated with a range of animal models of chronic inflammatory and neuropathic pain [69–72]. This is not surprising for GlyT1 inhibitors since reduction of the glial reuptake of glycine increases the synaptic concentration of the neurotransmitter and, consequently, the inhibitory tone in the spinal cord. The observation that GlyT2 inhibitors have a similar outcome rather suggests that a moderate inhibition of GlyT2 might increase synaptic level of glycine without a significant effect on its quantal release. This seems to be the case of ALX-1393, one of the best characterized GlyT2 inhibitors *in vivo* [73–80]. Intrathecal injection of ALX-1393 inhibits acute thermal and mechanical pain responses and is especially active in reversing sensitized responses, reducing mechanical allodynia in neuropathic and inflammatory pain models. Nevertheless, ALX-1393 has a poor penetrance through the blood–brain barrier and presents some cross-reactivity with GlyT1, therefore limiting its usefulness as a therapeutic tool [81]. In contrast, ORG25543 has an improved brain penetrance and specificity for GlyT2. The compound was effective at low concentrations in reducing allodynia in a model of neuropathic pain. However, at high concentrations ORG25543 was toxic and produced motor dysfunction, convulsions and spams [81]. Interestingly, lower affinity derivatives of ORG25543 seem to keep its efficacy as analgesic compounds without toxicity [81,82]. Recently, the phenoxymethylbenzamide compound GT-0198, a structurally novel and orally adminstrable GlyT2 inhibitor with excellent brain penetrance, showed a potent analgesic effect in the partial sciatic nerve ligation model of neuropathic pain, without side motor effects [83]. Finally, other compounds with promising profile for the development of GlyT2-based analgesics are derivatives of the endogenous fatty acid *N*-arachidonyl glycine (NAGly), especially abundant in the spinal cord. NAGly has analgesic properties in diverse experimental models and was shown to inhibit GlyT2 thereby enhancing glycinergic transmission at synapses [71,84,85]. As this compound also affects other proteins that might contribute to the analgesic effects (glycine receptor, cyclooxygenase-2, among others), a better understanding of its binding mechanism to GlyT2 may help to design novel compounds with the adequate pharmacodynamics profiles to enhance glycine neurotransmission without undesired motor side-effects.

## Concluding remarks

This review summarizes the data currently available regarding the involvement of GlyT2 in neuronal pathologies affecting both motor and sensory systems. GlyT2 expression determines, together with VIAAT, the glycinergic nature of a neuron and therefore it is a clue protein in the inhibitory function of these neurons in caudal areas of the brain. Glycinergic neurons control movement processes at the spinal level and genetic studies have revealed that mutations in the GlyT2 gene are associated with some forms of a rare myoclonic disorder, hyperekplexia. In the sensory system, glycinergic neurons control the excitability of the pain transmission pathways from the periphery to the brain. Chronic pain conditions are associated to the loss of this inhibitory mechanism. GlyT2 inhibitors have shown to restore inhibitory glycinergic function thereby providing analgesic effect. Recent advances in understanding the three-dimensional structure of SLC6 family members is expected to shed light on the mechanisms of neurotransmitter transport. Hopefully, the availability of these new tools and knowledge will be useful to develop drugs able to interfere with the glycinergic system and the associated functions.

## Abbreviations

EGFP: enhanced green fluorescent protein
HPX: hyperekplexia
GABA: γ-aminobutyric acid
GABAR: GABA receptor
GlyR: glycine receptor
KO: knockout
PKC: protein kinase C
PV: parvalbumin
TM: transmembrane
